# A scoping review of genetics and genomics research ethics policies and guidelines for Africa

**DOI:** 10.1186/s12910-021-00611-9

**Published:** 2021-04-02

**Authors:** Joseph Ali, Betty Cohn, Erisa Mwaka, Juli M. Bollinger, Betty Kwagala, John Barugahare, Nelson K. Sewankambo, Joseph Ochieng

**Affiliations:** 1 Johns Hopkins Berman Institute of Bioethics, 1809 Ashland Ave, Rm 208, Baltimore, MD 21205 USA; 2 Johns Hopkins Bloomberg School of Public Health, Baltimore, MD USA; 3 College of Health Sciences, Makerere University, Kampala, Uganda; 4 College of Business and Management, Makerere University, Kampala, Uganda; 5 Department of Philosophy, Makerere University, Kampala, Uganda

**Keywords:** Genetics and genomics research, Africa, Ethics, Policies, Laws

## Abstract

**Background:**

Genetics and genomics research (GGR) is increasingly being conducted around the world; yet, researchers and research oversight entities in many countries have struggled with ethical challenges. A range of ethics and regulatory issues need to be addressed through comprehensive policy frameworks that integrate with local environments. While important efforts have been made to enhance understanding and awareness of ethical dimensions of GGR in Africa, including through the H3Africa initiative, there remains a need for in-depth policy review, at a country-level, to inform and stimulate local policy development and revision on the continent.

**Methods:**

To identify and characterize existing ethics-related guidelines and laws applicable to GGR across much of Africa, we conducted a scoping review of English language policy documents identified through databases, repositories, and web searches. Thirty-six documents were included and coded using a framework that contained a range of themes across five analytical categories: (1) respect, (2) beneficence, (3) justice, (4) independent oversight, and (5) bans and prohibitions. Data analysis software (NVivo 12) was used to organize, code, and tabulate information according to document characteristics and topics. Illustrative examples of policy requirements were selected for inclusion.

**Results:**

Documents that met inclusion criteria spanned 20 years; published between 1996 and 2018, with the majority (58%) published after 2009. About two-thirds were denoted as “guidelines,” and slightly more than half were non-exclusive to GGR. Very few (six) country-level documents identified were specific to GGR. Requirements related to the principle of “respect” appeared most often across all documents, relative to other principles and processes. The most commonly stated ban was on reproductive cloning. Other prohibitions applied to germline editing, undue inducements in research, sample use for commercial purposes, employee mandatory DNA testing, fetal sex selection, stem cell use, eugenics, and research without public health benefits.

**Conclusions:**

Enforceable policies that are indispensable to the ethical conduct and review of GGR are either deficient or missing in many African countries. Existing international, GGR-specific ethics guidelines can be used to inform GGR policy development at a country-level, in conjunction with insight from country specific ethics committees and other local stakeholders.

## Background

Due to scientific advancements and the decreased cost of genomic sequencing technologies, genetics and genomics research (GGR) has increased in high-income countries [1, 2]. However, the growth and development of GGR is currently raising critical ethical concerns. For example, until recently, researchers within many less-resourced countries, including several across sub-Saharan Africa, who did not have access to up-to-date facilities, technologies, or training and who faced significant financial barriers, had to rely on genetic analyses being conducted outside of the countries of specimen origin. For many, this contributed to “a sense of mistrust and exploitation,” particularly among African researchers and research participants [3]. Even more so, foreign research teams would come to the continent, conduct research, and leave without much benefit to local populations [4]. At the same time, in a phenomenon that is described as “brain drain,” some accomplished African researchers—due to the challenging research environment—have left the continent in order to participate more fully in advancing the science of GGR [2]. In order to ensure equitable development of GGR and increase African populations’ chances for access to the preventive, diagnostic and therapeutic benefits, there is a need to identify and address these ethical challenges. Other common ethical challenges in GGR in Africa and elsewhere include issues related to informed consent [3–7], privacy and confidentiality (e.g., re-identifiability and data breaches) [3, 4, 7, 8], community engagement [7, 9, 10], data sharing [6, 8, 11, 12], and return of research results and incidental findings [6, 13–15].

A critical ingredient necessary to achieving the goal of meaningful representation and engagement of African researchers and populations in the global enterprise of GGR is the development of robust ethical, legal and policy frameworks [13]. The Human Heredity and Health in Africa (H3Africa) initiative was launched in 2010 in collaboration with the U.S. National Institutes of Health (NIH), the U.K. Wellcome Trust and the African Society for Human Genetics after a March 2009 meeting in Cameroon in order to begin studying genetic diversity in Africa. Part of H3Africa’s mission is to identify the scientific, ethical, and practical questions that accompany GGR in Africa [16]. Since 2010, H3Africa has published a white paper and other guidance documents to provide robust recommendations, and a comprehensive framework for ethical GGR in the African setting [16]. However, the guidance documents are generic and may not readily integrate with unique country level considerations. H3Africa has also sought to recognize the importance of generating local policies for GGR that are specific and responsive to different country contexts and, to the extent possible, reflective of the considerations and guidance provided by the H3Africa framework. However, at a country-level, according to a 2017 review, regulatory documents across much of Africa are “either absent, outdated, conservative or difficult to navigate” [6]. Where GGR policies and regulations do exist, it is currently unclear to what extent they reflect the recommendations and guidance that has been carefully articulated by the H3Africa Working Group on Ethics and other international organizations.

As part of a larger project to empirically establish guidelines for GGR in Uganda, we conducted a scoping review of the literature to evaluate existing guidelines, policies and laws related to the ethics and oversight of GGR, with a particular emphasis on documents developed by and for Africa. Our primary goal was to characterize the nature of existing governance documents that address the ethical oversight of GGR in Africa, and identify areas where those documents converge or diverge, as well as major gaps in policy frameworks.

## Method

This review sought to understand the nature of existing guidelines that are meant to inform the ethical oversight of GGR in Africa. We conducted our assessment by way of document collection, review, and synthesis, using a scoping review methodology. Scoping reviews are useful for assessing a research or policy area and identifying gaps [17]. The scoping review method that we utilized was proposed by Arksey and O’Malley (2005) which includes the following seven processes: (1) identify a research question, (2) identify relevant documents, (3) select documents, (4) chart data, (5) collate, (6) summarize and report results, and (7) consult with relevant stakeholders [17, 18].

### Data sources and search strategy

We conducted the search from October to December 2019. Documents were identified by reviewing existing databases and repositories—the U.S Office for Human Research Protections (OHRP) International Compilation of Human Research Standards, and policy documents published by H3Africa—as well as from a 2017 paper published by de Vries et al. that identified several GGR policy documents [6, 12, 19]. Several national documents, as well as documents authored by international organizations (“international documents”), were identified through these searches. For completeness, for each of the 54 African countries we also conducted a Google search for the following search strings: genetic AND guidelines AND [country name], genomic AND guidelines AND [country name], genetic AND guidance AND [country name], genomic AND guidance AND [country name], genetic AND policy AND [country name], genomic AND policy AND [country name], genetic AND law AND [country name], genomic AND law AND [country name], genetic AND code AND [country name], genomic AND code AND [country name], genetic AND act AND [country name], genomic AND act AND [country name]. In addition, we ran a search for websites of Ministries of Health in Africa and searched specifically for GGR policies on those government websites.

### Eligibility

Documents were eligible for inclusion if they constituted guidance, policy, or law addressing ethical aspects of GGR. Documents applicable to at least one country in Africa, or to Africa in general, were included. We excluded documents that were not written in English, or that only discussed biobanking of genetic information. We did not apply any date restrictions to the search.

### Literature screening

**Fig. 1:**
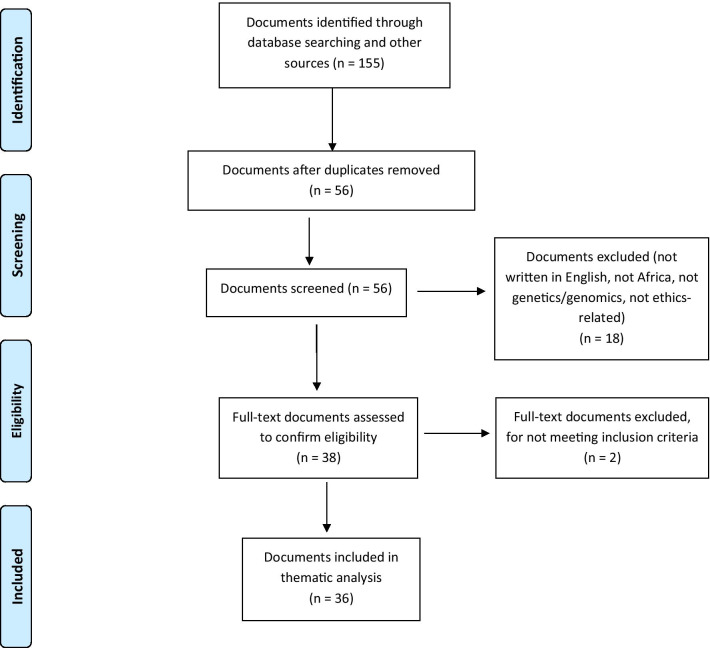
PRISMA flow diagram for document selection

We used Microsoft Excel to track documents and their basic characteristics. One hundred and fifty-five documents were initially identified as potentially eligible. Following de-duplication, 99 were excluded, leaving 56 for further screening. These documents, which appeared to be relevant based on their titles, were then reviewed to determine whether they met eligibility criteria. They were first screened for key words to identify whether they discussed genetics or genomics research in Africa or an African country. Next, they were screened to determine if they addressed ethics issues (as opposed to providing operational or technical guidance). This process resulted in exclusion of 18 additional documents, leaving 38 that were read again carefully, in full, to verify eligibility. This final review resulted in exclusion of two more documents for not meeting criteria, leaving 36 documents for data extraction and analysis (Fig. 1).

### Data abstraction, coding and analysis

Following collection of documents, we used a tracking spreadsheet to organize them into two major categories: documents that were GGR specific, and documents that were GGR non-specific. Within these two categories we further organized the documents by year of publication, level of organization, publishing institution, country, and level of authority. To support analysis of content within the documents, a codebook was created by all authors drawing heavily from the H3Africa *Ethics and Governance Framework for Best Practice in Genomic Research and Biobanking in Africa* [13]. We organized data according to codes (categories) and sub codes (topics). The codes covered five broad categories, three of which addressed core research ethics principles [20] including: (1) respect (topics: consent; sensitive to and respectful of African values, cultures, norms, or leadership; transparency; community engagement; and respect or dignity for participants), (2) beneficence (topics: potential harms or avoidance of harms; feedback of genetic results; benefits of research; capacity building; and responsiveness), and (3) justice (topics: managing data and samples; nondiscrimination; fairness and fair international collaboration).The fourth category, independent oversight, addressed institutional processes or systems that can support adherence to ethical principles (topics: ethics review; governance frameworks; and role of government). The last category, bans and prohibitions, was used to identify impermissible types of GGR. During the process of reviewing documents, the topics were iteratively revised for clarity and some new ones were added.

All documents that met the inclusion criteria were imported into NVivo 12 [21] and coded using the defined coding framework by one member of the research team (BC) whose coding was reviewed by two other members (JA, JMB) to ensure appropriate and consistent application of codes. Any coding discrepancies were discussed and resolved through consensus among the three members of the coding team. Frequencies, percentages and data visualizations were generated using NVivo 12 and Microsoft Excel.

## Results

Results are presented in three parts. First, we present an overview of the main characteristics of the guidance documents. Second, we highlight the distribution of categories and issues across the documents. Lastly, we present key findings from each of the five categories from our coding framework.

### Characteristics of documents reviewed

Thirty-six documents were included in our analysis. Their basic characteristics are detailed in this section and summarized in Table 1. Documents that met inclusion criteria spanned 20 years and were published between 1996 and 2018, with the majority (58%) published after 2009. Five documents were published in 2017, the most of any year, all of which were published by H3Africa. The 36 English language documents represented 14 of the 54 countries in Africa. Only four countries (Gambia, Malawi, Tanzania, and South Africa) published documents that exclusively addressed GGR [22–27]. The type of publishing organization varied. A majority (22) of the documents were published by a national institution such as a Ministry of Health, rather than by an international organization such as the World Health Organization (WHO).

**Table 1:** Basic characteristics of documents included in the analysis

Title	Year of publication	Level of organization	Publishing institution	Countries	Level of authority
**Exclusively addresses genetics and genomics research**					
HUGO Statement on The Principled Conduct of Genetics Research	1996	International	HUGO	International	Statement or Declaration
UNESCO Universal Declaration on the Human Genome and Human Rights Sect. 17	1997	International	UNESCO	International	Statement or Declaration
HUGO Statement on DNA Sampling Control and Access	1998	International	HUGO	International	Statement or Declaration
HUGO Statement on Gene Therapy Research	2001	International	HUGO	International	Statement or Declaration
Guidelines of the National DNA bank, The Gambia	2001	National	Government of The Gambia	Gambia	Guideline or Policy
HUGO Statement on Human Genomic Databases	2002	International	HUGO	International	Statement or Declaration
MRC Guidelines on Ethics for Medical Research, Reproductive Biology, and Genetic Research	2002	National	Medical Research Council of South Africa	South Africa	Guideline or Policy
Procedures and Guidelines for Access and Collection of Genetic Resources in Malawi	2002	National	National Research Council of Malawi	Malawi	Guideline or Policy
The Human DNA Regulation Act	2009	National	Government of Tanzania	Tanzania	Law or Act
Policy Requirements, Procedures and Guidelines for Conduct and Review of Human Genetic Research in Malawi	2012	National	National Health Sciences Research Committee	Malawi	Guideline or Policy
H3Africa Data and Biospecimen Access Committee Guidelines	2017	International	H3Africa	Africa	Guideline or Policy
H3Africa Decision Tree for Feedback of Individual Genetic Results	2017	International	H3Africa	Africa	Guideline or Policy
H3Africa Ethics and Governance Framework for Best Practice in Genomic Research and Biobanking in Africa	2017	International	H3Africa	Africa	Guideline or Policy
H3Africa Guidelines for Community Engagement	2017	International	H3Africa	Africa	Guideline or Policy
H3Africa Guidelines for the Return of Individual Genetic Research Findings	2017	International	H3Africa	Africa	Guideline or Policy
H3Africa Guideline for Informed Consent	2018	International	H3Africa	Africa	Guideline or Policy
Human Genetics and Genomics in South Africa: Ethical, Legal and Social Implications Consensus Study	2018	National	Academy of Science of South Africa	South Africa	Guideline or Policy
**Mentions genetics/genomics research**					
National Health Act	2004	National	Government of South Africa	South Africa	Law or Act
ISSCR Guidelines for the Conduct of Human Embryonic Stem Cell Research	2006	International	ISSCR	International	Guideline or Policy
National Biotechnology Authority Act	2006	National	Government of Zimbabwe	Zimbabwe	Law or Act
Guideline for Obtaining Informed Consent For the Procurement and Use of Human Tissues, Cells and Fluids in Research	2007	International	WHO	International	Guideline or Policy
National Code of Heath Research Ethics	2007	National	Ministry of Health	Nigeria	Law or Act
Guidelines for Ethical Conduct of Research Involving Human Subjects	2008	National	Ministry of Health	Sudan	Guideline or Policy
National Guidelines for Ethical Conduct of Research Involving Human Subjects	2008	National	Ministry of Health	Kenya	Guideline or Policy
Good Clinical Practice Regulations	2009	National	National Agency for Food and Drug Administration and Control	Nigeria	Law or Act
Rwanda Ministry of Health, National Research Ethics Committee Standard Operating Procedures	2009	National	Ministry of Health	Rwanda	Guideline or Policy
National policy for Science, Technology and Innovation	2011	National	Ministry of Higher Education, Science, and Technology	Angola	Guideline or Policy
Regulations Relating to Artificial Fertilisation of Persons (Regulations added to the National Health Act of 2003)	2012	National	Ministry of Health	South Africa	Law or Act
Policy Statement on Storage of Human Samples in Biobanks and Biorepositories in Nigeria	2013	National	National Health Research Ethics Committee of Nigeria (NHREC)	Nigeria	Guideline or Policy
The National Health Research Act	2013	National	Government of Zambia	Zambia	Law or Act
Guideline for Application to Conduct of Clinical Trials in Liberia	2014	National	Liberia Medicines and Health Products Regulatory Authority (LMHRA)	Liberia	Guideline or Policy
National Guidelines for Research Involving Humans as Research Participants	2014	National	Uganda National Council for Science and Technology (UNCST)	Uganda	Guideline or Policy
National Research Ethics Review Guideline	2014	National	FDRE Ministry of Science and Technology	Ethiopia	Guideline or Policy
Ethics in Health Research Principles, Processes and Structures	2015	National	Ministry of Health	South Africa	Guideline or Policy
WMA Declaration of Taipei on Ethical Considerations Regarding Health Databases and Biobanks	2016	International	WMA	International	Statement or Declaration
Research Registration and Clearance Policy and Guidelines	2016	National	Uganda National Council for Science and Technology	Uganda	Guideline or Policy

The documents spanned different levels of authority, ranging from more enforceable (e.g., laws or acts) to less enforceable (e.g., guidelines or policies, and statements or declarations). Laws and acts also tended to be broader in scope (6 out of the 7 laws/acts were broader documents). A majority (64%) of documents were denoted as “guidelines,” and slightly more than half (56%) were non-exclusive to GGR. For example, in the *National Research Ethics Review Guideline,* published in Ethiopia, GGR is embedded in the text that covers broader research ethics issues, whereas *Policy Requirements, Procedures, and Guidelines for Conduct and Review of Genetic Research in Malawi* is solely dedicated to GGR in Malawi [23, 28]. One document, *Human Genetics and Genomics in South Africa: Ethical, Legal, and Social Implications Consensus Study* was categorized as a guideline despite being labeled a “consensus study” as it provides ethical guidance for the South African government for GGR [29, 30].

### Distribution of categories and topics across documents

As shown in Table 2, requirements related to the principle of respect appeared most often across all documents, relative to other principles and processes that we coded. Thirty of the 36 documents referred to an application of respect, with the most common being consent which was referred to in 27 (75%) of documents. Within the category of consent, 8 documents discussed the content of the informed consent form, 7 documents discussed issues related to consent procedures, 6 documents discussed broad consent and the permissibility of broad consent, and 5 documents discussed withdrawal of consent. The second most common representation of respect across documents was reflected in provisions that emphasized the need for GGR to be sensitive to and respectful of African values, cultures, norms or leadership—noted across 19 documents. Additionally, of the documents that discussed respect, 15 discussed the importance of transparency, and 11 discussed sharing or disseminating study information, 9 discussed community engagement, and 7 discussed respect for participant rights and dignity.

**Table 2:** Distribution of categories and topics across documents

Category	Topic	Number of documents containing the category/topic n=36 n (%)
**Respect**		30(83)
	**Consent**	27(75)
	What should be included in the informed consent form	8(22)
	Consent procedures	7(19)
	Broad consent	5(14)
	When is broad consent permissible	6(17)
	Withdrawal of consent	5(14)
	Definition of broad consent	4(11)
	Other types of consent	4(11)
	Challenges of the informed consent process	3(8)
	Specific consent	3(8)
	Waiver of consent or no consent	3(8)
	Who determines type of consent	2(6)
	**Sensitive to and respectful of African values, cultures, norms and leadership**	19(53)
	**Transparency**	15(42)
	Sharing or disseminating study information	11(31)
	Conflict of interest	1(3)
	**Community engagement**	9(25)
	Goals of community engagement	5(14)
	Evaluation of community engagement efforts	4(11)
	Prerequisites for community engagement	4(11)
	Acknowledgement of implications of research on the community	3(8)
	Definition of community engagement	3(8)
	When in the research lifecycle should community engagement occur	3(8)
	Who should be engaged	2(6)
	**Respect or dignity for participants**	7(19)
**Beneficence**		26(72)
	**Potential harms or avoidance of harms**	24(67)
	Privacy, confidentiality, security	20(56)
	Minimizing harms	4(11)
	Vulnerable populations	3(8)
	Investigators should seek to understand existing/potential stigma to avoid further harm	2(6)
	**Feedback of genetic results**	17(47)
	Anticipating/ Planning for return of results	4(11)
	Procedural/Technical requirements before returning results	4(11)
	Challenges associated with providing individual genetic research results	2(6)
	Sharing aggregate results	2(6)
	Sharing individual results	1(3)
	**Benefits of research in general**	12(33)
	Benefit sharing	3(8)
	Benefits African population	3(8)
	**Capacity building**	11(31)
	What areas/topics is capacity building required	6(17)
	When is capacity building necessary	3(8)
	Who is responsible for capacity building	3(8)
	What is capacity building	2(6)
	**Responsiveness**	4(11)
**Justice**		22(61)
	**Managing data and samples**	20(56)
	Sample and data sharing	16(44)
	Data rights	8(22)
	Data use agreements	8(22)
	Material or sample rights	8(22)
	Material Transfer Agreements	7(19)
	Export of samples	6(17)
	Intellectual rights	5(14)
	Patents and IP	2(6)
	Sample and data storage	4(11)
	Sample and data storage (not specific to genetics)	6(17)
	Sample re-use	5(14)
	**Nondiscrimination**	13(36)
	**Fairness**	6(17)
	Fair distribution of benefits and burdens	3(8)
	Research should be relevant to the population under study	1(3)
	**Fair international collaboration**	2(6)
**Independent oversight**		24(67)
	**Ethics Review**	23(64)
	Role of ethics review	6(17)
	Process of ethics review	3(8)
	Substance of ethics review	1(3)
	**Governance framework**	7(19)
	**Role of government**	4(11)
**Bans and prohibitions**		12(33)
	Cloning	5(14)

The second most frequently coded principle across documents was beneficence, being represented in 26 (72%) of the 36 documents. Of the documents that mentioned beneficence, 24 discussed potential harms or avoidance of harms, and of these, a majority (20 documents) focused on issues of privacy, confidentiality or security involving genetic and genomic information. Several documents also mentioned feedback of genetic results; discussed in nearly half (17) of the documents. Only about one third (12) of documents explicitly provided guidance or articulated requirements related to realizing the benefits of GGR (e.g., sharing benefits with local communities). Finally, 11 documents discussed the need for GGR capacity building. Capacity building refers to the strengthening and supporting of research infrastructure for GGR and biobanking in Africa, including career support [13]. Other less frequently noted issues included special protections for vulnerable populations, raised in three documents.

We identified instances of the principle of justice across 22 (61%) of the documents. Issues discussed most frequently involved rights and interests related to data and samples (20 documents) and included content addressing the sharing of data and samples (16 documents), data rights (8 documents), data use agreements (8 documents), and material and sample rights (8 documents). The export of samples, and intellectual property rights were discussed in 6 and 5 documents respectively. Issues related to nondiscrimination were discussed across 13 documents. While 6 documents mentioned the need to ensure fair distribution of benefits and burdens of research and only 1 document mentioned the need to ensure research is relevant to local populations.

The fourth category in our coding framework is independent oversight, which was identified in 24 (67%) documents. Ethics review—one approach to operationalizing independent oversight—was referred to most often within this category: in 23 documents. Additionally, components of ethics review were broken down into the function or role of the ethics review (6 documents), ethics review procedures (3 documents), and the content of the ethics review (1 document). Lastly, how organizations should be governed, and the role of government were noted in 7 and 4 documents respectively.

Finally, bans or prohibitions (declarations of impermissible activities or types of GGR) were identified in 12 (33%) documents. The most common ban was on research that included reproductive cloning, which was prohibited in nearly half of the documents that mentioned a ban or prohibition (5 documents).

### Additional characteristics of provisions according to identified categories

#### Respect considerations

The ethics principle of respect was the most commonly identified category across guidance documents. Respect was discussed in the context of the following applications: consent; sensitivity to and respect for African values, cultures, norms, and leadership; transparency; community engagement; and respect or dignity for participants. In what follows, we describe how each of these appeared in the sampled documents.

##### Consent

Consent considerations appeared in various ways across the documents, including in defining elements that must be included in an informed consent form, describing required consent procedures, and characterizing the permissibility or features of broad consent.

Documents articulated required elements of informed consent in order to emphasize the importance of obtaining valid and comprehensive informed consent when conducting research with biological samples. However, only two documents (one from South Africa and one from H3Africa) noted that consent forms should include the possibility of incidental findings and should identify whether the research participant wants to receive those findings [27, 31].

The documents referenced consent procedures in different ways. Some documents emphasized that informed consent is not a single event (e.g., “The informed consent process for genomic studies starts prior to research, is on-going during the research and continues even after the research is over”) [27]. Other documents placed emphasis on this and the importance of verifying that consent procedures are indeed facilitating participant understanding (e.g., “Depending on the target population, H3Africa researchers may be required in some studies to demonstrate a participant’s understanding of research study elements during the consent process. This is particularly relevant to studies targeting populations who may be perceived as vulnerable to exploitation, like those with cognitive impairments.”) [32].

Broad consent—including its permissibility and constitutive elements—was noted in very few (6) documents. Three of the six documents mentioning broad consent were H3Africa documents [13, 14, 32] while the other three were documents from 2013 or later from Nigeria and South Africa. For example, *The Policy Statement on Storage of Human Samples in Biobanks and Biorepositories* in Nigeria stated that it “[supports] ‘broad consent’ at this time but not ‘blanket consent’ or ‘gifting’” [33]. However, the Zambian National Health Research Act remained silent on broad consent but does prohibit withdrawal of samples from individuals for any “unspecified future health research activity or unspecified storage.” [34].

Withdrawal of consent was specifically mentioned in a total of five documents—four international documents: H3Africa Framework for African Genomics and Biobanking, H3Africa Guideline for Informed Consent (Third Edition), Human Gene Organization (HUGO) Statement on DNA Sampling Control and World Medical Association (WMA) Declaration of Taipei on Ethical Considerations Regarding Health Databases and Biobanks [11, 13, 32, 35] and one national document: *Human Genetics and Genomics in South Africa: Ethical, Legal, and Social Implications* [27]. The documents indicate that researchers should make it possible for participants to withdraw consent, but also state that withdrawal of consent is not possible in circumstances where samples have been permanently de-identified or if data have already been published [13, 32].

Of the eight documents that do not mention consent, two were international documents (one of which was a decision support tool for feedback of individual genetic results), and six were national documents (two of which were national laws).

##### Sensitive to and respectful of african values, cultures, norms or leadership

Some applications of respect that appeared in the documents were coded as “sensitive to and respectful of African values, cultures, norms or leadership.” This code spans many topics, and thus appeared in various forms. Almost all of the international documents (11 of 14) recommended being sensitive to and respectful of African values, cultures, norms, or leadership. However, only 8 of the 22 national documents were found to address this requirement explicitly. Additionally, this requirement was generally noted when discussing consent or feedback of results to participants, where emphasis was placed on acknowledging communitarian norms common in parts of Africa. When international documents discussed this issue, it was represented in very similar language across documents (e.g., communication between researchers and participants, and between researchers and communities should be “sensitive to their social and cultural context”) [36]. National documents also tended to operationalize this concept in terms of cultural and epistemic sensitivity—for example, the *National Guidelines for Research Involving Humans as Research Participants* published by the Uganda National Council for Science and Technology (UNCST), notes that “indigenous knowledge of the community should be recognized” [37]. They also identified specific local groups that should be involved in GGR, such as the Ministry of Health, and local collaborators and institutions [23].

##### Transparency

Provisions related to transparency were identified across 15 documents. Often this appeared as an emphasis on the importance of complete disclosure of relevant information during consent and feedback of genetic results, including clear articulation of available options. For example, *Guidelines of the National DNA Bank*, published by the Federal Ministry of Health in Gambia, explains that “researchers should state in their proposals which results, if any, they will make available to research subjects. The research subjects have a right to know, but can decide that they do not want to know,” [22]. Indeed, 11 of the 15 documents discuss sharing or disseminating study information in an appropriate manner.

##### Community engagement

Community engagement and its applications were mentioned mostly in international documents and in only three national documents—those from South Africa, Tanzania, and Malawi. For instance, the Human DNA Regulation Act (2009) from Tanzania states that Technical Committees consisting of individuals from public and private sectors (e.g., representatives from the National Institute for Medical Research, a biotechnology department from an in-country University, the Department of Social Welfare, etc.) should reach out to members of communities with “relevant expertise” when necessary in order to make decisions about the ethics of GGR in Tanzania [25], while the *Policy Requirements, Procedures and Guidelines for the Conduct and Review of Human Genetic Research in Malawi* emphasizes the need for “stakeholder consultations” that “fits [Malawi’s] own context” [23].

In contrast to national documents, international documents were found to explicitly use the term “community engagement” in their guidelines [9, 13, 14, 32, 38, 39]. Indeed, H3Africa has developed a document that exclusively provides recommendations for community engagement in GGR and defines it as “a process of informing, consulting and actively involving relevant communities that have a legitimate interest in the research process,” [9]. Other H3Africa documents offer bulleted recommendations of how to engage the community in GGR and the importance of doing so.

##### Participant rights and dignity

Lastly, an issue that was emphasized the least under the category of respect was respect for participant rights and dignity. This issue was referenced in a more abstract way (e.g., by stating rights claims of the participants). Documents tended to emphasize participants and citizens’ rights at large. For example, one document noted that “everyone has a right to respect for their dignity and for their rights regardless of their genetic characteristics” [5].

#### Beneficence considerations

We identified standards and requirements related to the principle of beneficence within 26 documents (72%). Beneficence was discussed in the context of the following issues: potential harms or avoidance of harms, feedback of genetic results, benefits of research, capacity building and responsiveness.

##### Potential harms or avoidance of harms

Potential harms or avoidance of harms associated with GGR was the most noted issue under the category of beneficence, appearing across 67% of all documents. The issue arose most in documents that were classified as international, in guidelines, and in documents that were limited to GGR. Some documents emphasized stigma, while others emphasized collateral or secondary harms that accrued outside the individual research participant, such as familial, communal, or ethnic harms (e.g., harm to one’s ethnic group). Twelve documents were coded as not directly addressing harms or harm avoidance, with only one of those 12 being an international document—a decision support tool for feedback of individual genetic results [31]. The other 11 documents that do not mention this issue include both national guidelines/policies and laws/acts.

The most common representations of potential harms from GGR related to informational harms and therefore documents emphasized the importance of privacy, confidentiality, and data and sample security. Similar language was used across documents to signal this. For example, a Gambian guidelines states that “the protection of confidentiality has the highest priority,” [22], and a South African guideline states that “confidentiality of all medical information is essential, and this is particularly the case with genetic registers…,” [26]. The importance of de-identifying samples is emphasized to protect privacy, but few documents note the possibility of re-identification, which can diminish protections. The *Policy Statement on Storage of Human Samples in Biobanks and Biorepositories, the National Health Research Ethics Committee of Nigeria* notes this possibility by not only outlining how there could be a privacy breach, but also differentiating between anonymized and de-identified data (e.g., “De-identified data is not necessarily anonymized data because the personally identifying information may be able to be re-associated with the data at a later time. Anonymized data is a particularized subset of de-identified data” [33].

##### Feedback of genetic results

The next issue in terms of frequency of appearance under the category of beneficence was feedback of genetic results. A majority of documents (9 documents) that discussed feedback of genetic results were published by national institutions. The way in which feedback of genetic results was discussed in the documents varied. For example, some documents mentioned whether or not information should be shared with family members (7 documents), while others required that information be shared in a culturally sensitive way and that communities should be engaged accordingly. However, one GGR policy states that individual results should not be given back to study participants [28]. Lastly, little guidance was provided with respect to handling incidental findings or using genetic counselors when returning results.

##### Benefits of research

Half of the documents that mention the benefits of GGR were national documents. Those documents emphasized benefits of research for the country and the benefits of research (e.g., positive public health outcomes, or individual health benefits) for research participants and their communities. Additionally, two of the international documents articulated the benefits of GGR for the world at large [13, 36].

##### Capacity building

Capacity building was discussed in both international and national documents almost equally; however, no documents provided a formal definition of capacity building (sometimes referred to as capacity development). One document articulated the targets for capacity building (i.e., “It is of key importance that genomic research and biobanking conducted in Africa lead to substantive building of research capacity, including both human resources and research infrastructure” [13]. When capacity building was necessary and who was responsible for capacity building was also rarely mentioned. Capacity building was discussed similarly across the documents, using similar language that emphasized the importance for capacity building and that researchers in Africa, or within a specific country, should be trained to conduct GGR in their country.

#### Justice

Overall, justice considerations were infrequently identified across the documents. However, issues that intersected with the principle of justice were identified in the context of guidance and policies related to the management of data and samples. Topics that were coded within this included patents and intellectual property, and sample re-use. Issues of nondiscrimination, fairness, fair international collaboration were less commonly mentioned, though they did appear relatively frequently in H3Africa and HUGO documents.

##### Managing data and samples

We categorized data and sample management as an application of the principle of justice due to the connection between good governance of data and samples and principles of fairness in access. Within this issue, many topics are drawn out that discuss the components of data and sample management such as sharing, which was referred to the most, following rights, agreements, and storage. Documents that refer to the sharing of data and samples tended to be concerned with the historical exploitation of African researchers, intellectual property, data, and the African population in general throughout the GGR process. Furthermore, documents that referred to the management of data and samples were overwhelmingly guidelines or policies and published by national entities. Another trend that we noted was that over time, there was an increase in the discussion of data and sample management and its various components. The issue is mostly referred to in documents published from 2009 to 2018. Lastly, the documents that mention this issue tended to be solely concerned with data, biobanking, or samples, and mentioned one of these terms in the title, rather than documents that were concerned with ethical health research in general.

##### Nondiscrimination

Nondiscrimination was mentioned in 7 guideline documents and 5 statements or declarations. Furthermore, documents that were limited to ethical discussion of GGR referred to nondiscrimination more than documents that were broader in scope. When documents did refer to discrimination, they tend to also use the term stigma, and the potential harms associated with GGR resulting in discrimination. For example, some international documents specifically noted the importance of offering protections in research and recommended providing counseling to research participants due to the potential stigma and discrimination that could emerge from GGR among individuals from Africa [9, 32, 35, 39].

#### Independent oversight

The fourth category applied in the identified documents is independent oversight. Independent oversight includes institutional processes or systems that facilitate adherence to research ethics principles. This includes ethics review of protocols, deployment of governance frameworks, and articulating the role of government in oversight.

##### Ethics review

Ethics review was often noted in international documents and was frequently discussed as an important safeguard when samples are being collected from individuals for research. Two H3Africa documents suggested that ethics committees in various African countries may be inadequately equipped to review GGR, and experts may need to be consulted by ethics committees to support review processes [13, 14]. This was connected back to the need for capacity strengthening, but in this case with a focus on the training needs of research ethics committees [13]. Additionally, five documents, including three national documents, indicated that there must be ethics review and approval in order to conduct secondary analyses of samples [13, 27, 28, 32, 37].

##### Governance framework and role of government

The issue of governance is mostly noted in international documents and is often referred to in the context of management of samples. For example, documents use language such as “an appropriate governance framework for genomics resources should outline the policies (data and sample sharing…) regulatory matters, as well as entities… that deal with applications to access and use genomic resources,” [27]. With respect to the role of government, some documents addressed the role of government in regulatory processes involving GGR, including defining the jurisdiction of national ethics committees and other legal regulatory bodies [25–27].

### Bans and prohibitions

The most commonly stated ban was one on reproductive cloning [5, 23, 27, 34, 40]. Others bans or prohibitions applied to germline editing [23, 41], undue inducements in research [25, 36], sample use for commercial purposes [23, 25], employee mandatory DNA testing [25], fetal sex selection [26], stem cell use [26, 27, 40], eugenic purposes [23], and research without public health benefits [23]. One such example from the literature includes Nigeria’s *Good Clinical Practice Regulations* where the National Agency for Food and Drug Administration and Control states that “No gene therapy trials shall be carried out which result in modifications to the participant's germ line genetic identity,” [41]. Additionally, *Malawi’s Policy Requirements, Procedures and Guidelines for the Conduct and Review of Human Genetic Research*, declares that “where there is no therapy, and prevention strategies are unknown or unproven, genetic testing is unethical and is herein prohibited as such research cannot offer a therapeutic and/or public health value,” [23]. No other document mentioned a prohibition of genetic testing in its entirely, absent benefit to participants.

## Discussion

Genetics and genomics research have the potential to make advances against a wide range of communicable and noncommunicable diseases affecting populations within low-resourced countries, including across Sub-Saharan Africa. Successful realization of these benefits partly depends on identification and establishment of local (country) ethics norms and processes. By its nature, especially given the amount and type of information that can be revealed about research participants (as individuals, families, communities or races) GGR raises significant and unique ethical concerns. Consequently, in order to effectively address these and related concerns, formal guidelines and regulations are needed to provide clarity for researchers and research oversight entities, as well as increase public trust and confidence in GGR are needed.

The majority of documents identified by this scoping review constituted guidelines or policies published by national entities that referenced special provisions for GGR in the context of a broader set of requirements related to the appropriate conduct of research. Very few (six) country-level documents identified were specific to GGR. International documents were found to be more likely to address GGR exclusively; however, the majority of international documents were developed by one organization—the H3Africa Consortium. Although an important contribution, international guidance documents do not lend themselves readily to local enforcement at a country level. Clearly, a tension exists between development of uniform multi-national standards and guidelines for the ethical conduct of GGR, and development of local policies that align with existing systems of governance and oversight, as well as local norms and values. A 2013 study concerned with GGR in Nigeria noted one of the greatest difficulties in GGR regulation as: “harmonizing the different laws and customs that apply to genomic research in different jurisdictions… [and] [meeting] the needs of Nigerian communities and research participants” [1]. Policy reviews, such as the present, can be helpful in guiding deliberation regarding localization of provisions within international frameworks and identifying variations across jurisdictions that might suit a country’s needs. There is also value, as discussed further below, in identifying gaps in policy.

Different types of governance documents applicable to GGR in Africa serve different functions. Whether a document is a framework, guideline, statement, or law reflects not only the level of enforceability, but also often the degree to which there is consensus (professionally or socio-culturally) regarding the ethical implications of advances in the field of genetics and genomics. A framework or guideline, for example, can be more readily established and modified than a law, and can therefore remain more closely connected to scientific developments and capabilities. Scientific innovation moves at a pace that often cannot be matched by the law. Statements, which are typically international, can drive broader change in policy or practice, or preserve and advance global interests. Laws can play an important role in setting the outer boundaries of acceptable or unacceptable research, reducing societal uncertainty, and governing uses of genetic information that present significant risk to individuals or groups, especially uses that are inconsistent with basic rights. The law must do so in a manner that is fair and clear, taking into account the balance of various stakeholder interests to promote public benefit and reduce harm. While ethics and law overlap, and the law may generally support or be supported by ethics norms, it also operates within procedural and jurisdictional limits.

While recently published policy frameworks, such as that from South Africa [27], are noteworthy for their comprehensive coverage of the topic, a gap exists with respect to publication of country-level GGR-specific policies and laws across a majority of the African countries in our review. Others have noted that national policies are important to the management of genetic/genomic data and samples [42]. Due to historical and ongoing concerns about exploitation through research in Africa, especially with respect to data and sample management and the increased ease in the ability to share data and samples, the gap is particularly noteworthy [2]. Further, those involved in developing or updating GGR policies should consider harmonization with other existing or emerging frameworks for governance of biobanks and oversight of emerging data science capabilities, including the use of artificial intelligence and machine learning in “big data” research. Data science and biobanking have far wider application, raise additional ethics and regulatory issues, and deserve focused scholarly and policy attention; but as “facilitators” of GGR, it would be short-sighted not to consider opportunities for mutual and integrated strengthening of ethics and regulatory frameworks across these areas. Such opportunities are likely to increase in the coming years, especially given recent investments by the U.S. National Institutes of Health in the *Harnessing Data Science for Health Discovery and Innovation in Africa* (DS-I Africa) program [43]. Here we emphasize that although policy documents are imperative to supporting ethical conduct of GGR in Africa, this is only one piece of the puzzle: securing local sources of funding for GGR, providing ethics committees with necessary resources, and advancing ethics training for researchers and others is also critical. However, due to the gaps and lack of specificity in national guidance documents, ethics committees in many countries may feel ill prepared and indeed may disparately and inconsistently formulate determinations regarding management of data and samples [42]. The limited coverage of capacity building across documents is especially noteworthy, given aforementioned concerns about “brain drain” on the continent.

Furthermore, our review found that H3Africa documents tended to include more emerging themes—such as broad consent, data sharing, community engagement, respect for African leadership, and governance of biobanks—compared to other documents. Documents that represented national perspectives did not always address these elements. This is not particularly surprising given that most H3Africa documents emerged within five years prior to this review, that the organization had access to a wealth of expertise in ethics and governance of GGR, and that it facilitated interaction of leaders from multiple African countries to work together to create state of the art guidelines and frameworks [13]. H3Africa has had a tremendous impact on the development of ethics guidance across a range of domains, within the consortium. Indeed, its focus on fostering African (professional) leadership in the area of GGR is particularly noteworthy and lessons can be drawn from experiences with this model to potentially foster greater integration of similar principles in *national* policies. At the community level, practices and procedures such as those described within emerging models for community engagement and oversight in research can serve to help integrate community values in GGR in way that enhances its relevance and ethical quality [29, 30]. Policy development and revision can proceed at a local level with these frameworks in mind. A challenge for the future will be to do this, while maintaining some degree of communication and coordination to advance discussions of the ethical, legal and societal implications of GGR across geopolitical boundaries on the continent, given that the H3Africa network is only funded through 2021 [44]. Other organizations, such as the African Academy of Sciences, could be resourced to continue to advance this goal.

A limitation of this scoping review is that within our search we only considered documents available in English. We recognize that there may have been documents that met the rest of our inclusion criteria, but written in other languages. Additionally, we acknowledge that our focus was on documents that *explicitly* addressed GGR, which means that we did not address the content of other broader policies or guidelines that could have applied to GGR, simply by virtue of it being a form of research. We also note that our search strategy, which included identifying documents from existing compilations and internet searches using key words, could have missed documents due to limitations of the databases searched, the key words used, or the fact that the search did not include collection of “offline” documents. Furthermore, because we didn’t include scholarly literature in our scoping review, we may have missed explications of guidance documents that may have expressed how these guidance documents have been implemented, studied or interpreted. Finally, there is one document related to data sharing and data accessibility published by H3Africa that was not included because it was published after our search was completed [8]. Nonetheless, we believe it important to periodically evaluate the characteristics of existing formal governance documents, such as those reviewed in this study (as they exist “on their face”) to identify areas where those documents intersect, as well as major gaps in coverage.

## Conclusion

Our review found that local, enforceable policies which are indispensable for the increase of ethical GGR are either deficient or missing in many African countries. We suggest that existing international, GGR-specific, ethics guidelines be used to inform country-level GGR policy development, in conjunction with insight from country specific ethics committees and other local stakeholders. H3Africa’s comprehensive ethical framework can be used as a baseline for each African country. Each country can adopt relevant components of this framework and contextualize them within their local policy environments combined with relevant evidence generated using inclusive participatory approaches in order to create country-specific, enforceable frameworks. This process will ultimately promote the conduct of ethical GGR and will contribute to maximizing benefits for African researchers and populations.

## Data Availability

Data sources are available on request to the corresponding author. The coding framework applied in this study is provided with the manuscript.

## Abbreviations

GGR: Genetics and Genomics Research
NIH: National Institutes of Health
HUGO: Human Gene Organization
H3Africa: Human Heredity and Health in Africa
OHRP: Office for Human Research Protections
UNCST: Uganda National Council for Science and Technology
WHO: World Health Organization
WMA: World Medical Association
